# EXP2SL: A Machine Learning Framework for Cell-Line-Specific Synthetic Lethality Prediction

**DOI:** 10.3389/fphar.2020.00112

**Published:** 2020-02-28

**Authors:** Fangping Wan, Shuya Li, Tingzhong Tian, Yipin Lei, Dan Zhao, Jianyang Zeng

**Affiliations:** ^1^ Institute of Interdisciplinary Information Science, Tsinghua University, Beijing, China; ^2^ Machine Learning Department, Silexon AI Technology Co. Ltd., Nanjing, China

**Keywords:** synthetic lethality, L1000 gene expression profiles, machine learning, semi-supervised neural network, target identification

## Abstract

Synthetic lethality (SL), an important type of genetic interaction, can provide useful insight into the target identification process for the development of anticancer therapeutics. Although several well-established SL gene pairs have been verified to be conserved in humans, most SL interactions remain cell-line specific. Here, we demonstrated that the cell-line-specific gene expression profiles derived from the shRNA perturbation experiments performed in the LINCS L1000 project can provide useful features for predicting SL interactions in human. In this paper, we developed a semi-supervised neural network-based method called EXP2SL to accurately identify SL interactions from the L1000 gene expression profiles. Through a systematic evaluation on the SL datasets of three different cell lines, we demonstrated that our model achieved better performance than the baseline methods and verified the effectiveness of using the L1000 gene expression features and the semi-supervise training technique in SL prediction.

## Introduction

Two genes are considered a synthetic lethal (SL) pair if perturbation of both genes induces a defect in cell viability, while perturbation of either gene is not harmful to cell survival (Boone et al., 2007). Different types of perturbations were considered to trigger SL in previous studies, including knockdown, knockout, mutation, aberrant gene expression, copy number variation, and drug treatment (Whitehurst et al., 2007; Jerby-Arnon et al., 2014; Han et al., 2017; Sinha et al., 2017). Studying synthetic lethal interactions may help gain novel insights into target identification. Many cancer cells carry specific mutations in one gene (e.g., a tumor suppressor gene) of a synthetic lethal pair, and thus its synthetic lethal partner becomes a promising drug target (O'Neil et al., 2017). For example, the known synthetic lethal interactions between the tumor suppressor gene *BRCA1/2* and the drug target gene *PARP1* can be used to selectively kill cancer cells by triggering fatal DNA damages (Bryant et al., 2005; Farmer et al., 2005). To this end, PARP1 inhibitors have been approved to treat certain types of *BRCA*-mutated cancers (Fong et al., 2009).

SL gene pairs can be experimentally screened by developing double-knockout strains in model organisms and human cell lines. The synthetic lethality network in yeast has been well constructed using synthetic genetic arrays (SGA) (Tong et al., 2001) and diploid synthetic lethality analysis with microarrays (dSLAM) (Pan et al., 2007). Nearly one million gene pairs covering 90% of the whole yeast genome were screened in a recent study (Costanzo et al., 2016). Compared to yeast strains, which can undergo sexual reproduction to generate double-knockout offspring from parents bearing different single knockouts, it is more challenging to develop double-knockout human cell lines in an efficient manner. Thus, a relatively low number of human gene pairs (about hundreds or thousands) can be screened by RNA interference (Whitehurst et al., 2007; Barbie et al., 2009) and CRISPR-Cas9 (Shen et al., 2017; Han et al., 2017) based double-knockout experiments. Due to the difficulty in the establishment of large-scale double-knockout systems in human cell lines, the currently screened gene pairs only account for a small fraction of all possible combinations of human genes.

To overcome the current difficulty in experimental screen and generate more SL interactions in human, computational methods have recently been proposed to predict novel human SL pairs recently. The most direct idea is to leverage the abundant SL pairs characterized in yeast to infer human SLs through ortholog mapping (Deshpande et al., 2013; Wu et al., 2013; Srivas et al., 2016). The application of these methods was limited, as a large number of human genes do not have evolutionarily close yeast orthologs. Network-based methods predict human SLs through analyzing the protein-protein interaction (PPI) networks, metabolic networks, or signaling pathways (Folger et al., 2011; Kranthi et al., 2013; Zhang et al., 2015; Apaolaza et al., 2017). Statistical methods were also developed to identify SL gene pairs from human cancer cells based on the principle that the perturbations (*e.g.*, mutation, aberrant gene expression, and copy number variation) of both SL genes should be subject to negative selection and exhibit a mutually exclusive pattern (Jerby-Arnon et al., 2014; Srihari et al., 2015; Jacunski et al., 2015; Sinha et al., 2017; Lee et al., 2018). Besides, there exist several machine-learning-based approaches for predicting SL gene pairs. Most of these approaches learn from the adequate amount of supervised information of yeast (Wong et al., 2004; Pandey et al., 2010; Li et al., 2011). Only a few machine learning methods for predicting human SLs were developed. For example, Das et al. used a Random Forest classifier with multi-omics features (*e.g.*, differential expression, expression correlation, mutual exclusivity and shared pathways) to predict SL pairs in human cancer (Das et al., 2018); and Liu et al. proposed a logistic matrix factorization model regularized by the PPI similarity network and the gene ontology (GO) semantic similarity network to predict SL pairs (Liu et al., 2019).

Although a number of SL interactions are conserved in humans, most of them are only observed in specific cell lines or tissues (Ryan et al., 2018). A recent study detected SL pairs in three cell lines and found that only about 10% of SL interactions were shared by two cell lines, and no SL pair was identified in all the three cell lines (Shen et al., 2017). Despite the extensive applications of the above computational methods in SL prediction, most of them make predictions for the human genetic network without considering the cell line or tissue context. Although one of the aforementioned methods (Das et al., 2018) can predict SL in different human cancer types, it is difficult to directly apply this method to cell lines, as the homogenous genetic background of cell lines cannot provide enough mutation-related omics data. To provide a feasible tool for capturing the unique SL interaction networks for individual cell types, we aim to develop a computational method to learn from the experimentally measured SL interactions through considering the cell-line specific genetic information.

In this paper, we have proposed a novel computational method, EXP2SL, to predict cell-line specific SL interactions in human. The cell-line specific gene expression profiles resulting from the shRNA knockdown experiments in the LINCS L1000 project (Subramanian et al., 2017) were used to capture the information of cell-line specific genetic background. Since the available labeled data in single cell lines are limited, a semi-supervised objective function is used to exploit the large amount of unlabeled data. Tested on the combinatorial CRISPR-Cas9 perturbation-based SL datasets in three different cell lines, our model showed competitive prediction ability compared to the baseline methods. We also verified the effectiveness of the features derived from the L1000 gene expression profiles and the semi-supervised objective function. Furthermore, we evaluated the importance of each gene included in the L1000 gene expression profiles and found that the cell viability related functions were enriched among the top attributing genes.

## Methods

### Data Processing

#### The L1000 Gene Expression Profiles

The LINCS L1000 project (Subramanian et al., 2017) measured the expression levels of 978 landmark genes under different perturbations (*i.e.*, shRNA or compounds) and control conditions (*i.e.*, empty vectors or solvents) in different human cell lines. Here, we used the gene expression profiles resulting from shRNA perturbations to construct the features of the corresponding shRNA target genes, which were 978-dimensional vectors.

Specifically, the raw data from the LINCS L1000 project were preprocessed based on the pipeline in the original paper (Subramanian et al., 2017) with minor modifications; We first directly obtained the Level 3 data from L1000, which contained the quantile normalized gene expression profiles. The shRNA profiles perturbed after 96 hours were used, as the data amount for this time point was the largest. Based on this dataset, we calculated the z-score for each dimension of a shRNA perturbed profile *x*∈*ℝ* ^978^ by

(1) $$z=\frac{x-median\left(V\right)}{1.4826*MAD\left(V\right)},$$

where ***z*** is a 978-dimensional z-score of the shRNA perturbation profile ***x***, ***V*** is the set of vector control profiles from the same plate, *median(**V**)* and *MAD(**V**)* stand for the median value and the median absolute deviation of ***V***, and 1.4826 is a scaling factor to make the resulted z-scores close to normal distribution. Notably, in the original L1000 preprocessing pipeline (Subramanian et al., 2017), the control profiles were replaced by all the profiles on the plate, called population control. Here, we argue that this data preprocessing scheme may cause a biased control distribution due to the specific perturbation design. Thus, we use the expression levels treated with empty vectors as the control for the shRNA perturbed profiles.

For each gene, typically more than one types of shRNA were designed to knock down the expression of the corresponding gene product. To eliminate the off-target effects of shRNAs and obtain a robust signature for each single gene, the z-scores obtained from the replicated trials of the same shRNA were first processed using an algorithm with L1000 Level 5 data (Subramanian et al., 2017), then the same protocol was used to reduce the shRNAs targeting the same gene. More specifically, the z-scores were weighted and averaged according to the Spearman correlations to obtain a final 978-dimensional L1000 gene expression profile for each gene, which was then used as the input gene features for our model and other baseline models.

#### SL Labels

The SL labels in our datasets were constructed from the CRISPR double-knockout experiments performed in human cell lines (Shen et al., 2017; Zhao et al., 2018; Najm et al., 2018). A recently proposed computational approach called GEMINI (Zamanighomi et al., 2019) was used to identify SL interactions from the combinatorial CRISPR perturbation based cell viability studies. We adopted the GEMINI scores to select the positive and negative SL pairs for constructing our datasets. In particular, for each cell line, positive SL pairs were selected from gene pairs satisfying two criteria: 1) GEMINI “strong” scores larger than zero, which indicates the existence of the synergic lethal effect, and 2) GEMINI “strong” scores ranking among top 5%, to reduce the potential false positives. The main reason for choosing this threshold is that the top 5% gene pairs were considered as “the most significant hits in each screen” in the GEMINI paper (Zamanighomi et al., 2019). To more thoroughly evaluate the performance of our method, we also tested another threshold (*i.e.*, 10%) for choosing the positive SL pairs (**Tables S1-S2**). Negative SL pairs were those gene pairs satisfying 1) a GEMINI “strong” score less than zero, which means that there exists no synergic lethal effect between these two genes, and 2) a GEMINI “strong” score among the bottom 50%, to remove the potential false negatives. The gene pairs that were not selected as positive or negative SL pairs were considered as unknown pairs. Finally, cell lines with adequate numbers (>100) of gene pairs with both SL labels and L1000 gene expression profiles, including A549, A375, and HT29, were used in our study. The numbers of training samples for the cell lines are summarized in **Table 1**.

**Table 1:** Number of labeled training samples for each cell line.

	A549	A375	HT29
Positive SL gene pairs	126	18	18
Negative SL gene pairs	1106	44	123
Total	1232	62	141

### The Workflow of EXP2SL

**Figure 1:**
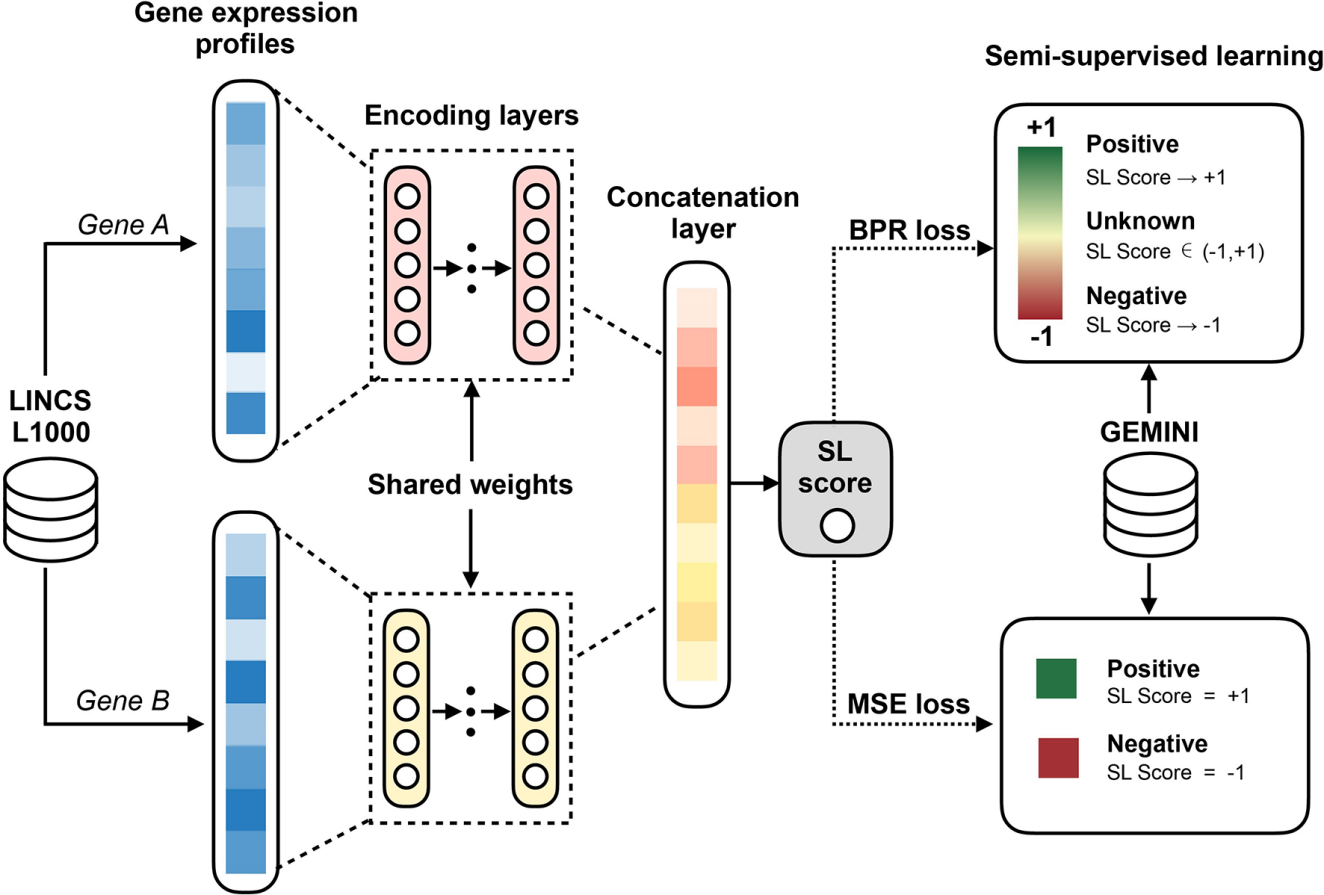
Workflow of the EXP2SL model. For a pair of gene, their L1000 gene expression profiles derived from knockdown conditions are the inputs of the encoding layers. Then, the updated features for both genes in a given pair are concatenated to predict the confidence score of being an SL pair by a linear combination. In addition, a semi-supervised objective function is used to train the model parameters, which aims to utilize the information from both known (positive and negative) and unknown SL gene pairs.

The basic idea of our EXP2SL model is to extract useful information from the L1000 expression profiles to accurately predict cell-line specific SL interactions. To achieve this goal, a semi-supervised objective function was designed to fully exploit the large amount of unlabeled data (**Figure 1**).

#### The Network Architecture of EXP2SL

For a given cell line, suppose that there are *N* genes (marked as the indices 1, 2,…, *N*) with measured shRNA data from the LINCS L1000 project (Subramanian et al., 2017). The corresponding L1000 gene expression profiles can be represented as a set of feature vectors ${\left\{f_{i}\in {\mathbb{R}}^{978}\right\}}_{i=1}^{N}$.

For a given cell line, our model first encodes the gene features through *E* sequential fully-connected layers, that is,

(2) $$h_{i}^{e}=ReLU\left(W_{encoder}^{e}h_{i}^{e-1}+b_{encoder}^{e}\right),e=1,2,\ldots ,E,i=1,2,\ldots ,N,$$

where $h_{i}^{0}=f_{i}$, *ReLU*(*x*) stands for the rectifier linear activation function *ReLU*(*x*) = *max*(0,*x*), $W_{encoder}^{1}\in {\mathbb{R}}^{d\times 978},W_{encoder}^{e}\in {\mathbb{R}}^{d\times d}(e=2,\ldots ,E)$, and $b_{encoder}^{e}\in {\mathbb{R}}^{d}(e=1,\ldots ,E)$ denote the learnable parameters (*d* is the dimension of the hidden layers).

After *E* encoding layers, the updated gene features ${\left\{h_{i}^{E}\right\}}_{i=1}^{N}$ are then used to predict SL interactions. More specifically, for a gene pair (*i*, *j*), *i*, *j* = 1,2,…, *N* and *i* ≠ *j*, a confidence score is calculated through a linear layer to predict the potential of SL interaction between this gene pair, that is,

(3) $$s_{i,j}=\frac{1}{2}\left(W_{out}\left[h_{i}^{E},h_{j}^{E}]+W_{out}[h_{j}^{E},h_{i}^{E}\right]\right)+b_{out},$$

where ***W***_*out*_∈*ℝ* ^1×2*d*^ and ***b***_*out*_∈*ℝ* stand for learnable parameters. Note that the pairs (*i*, *j*) and (*j*, *i*) are equivalent to each other, so we calculate the average prediction scores of concatenations of $\left[h_{i}^{E},\;h_{j}^{E}\right]$ and $\left[h_{j}^{E},\;h_{i}^{E}\right]$ to obtain the equivalent prediction results for input pairs (*i*, *j*) and (*j*, *i*).

#### The Semi-Supervised Objective Function

As described in SL Labels, the gene pairs with different SL labels can be classified into positive, negative, and unknown sets, denoted as *P*, *N*, and *U*, respectively. Here, we designed a semi-supervised loss function that utilizes information from all three sets to optimize the parameters of our model. More specifically, our loss consisted of three parts:

The first part of our objective function is the mean squared error (MSE) of positive and negative samples, calculated as

(4) $$L_{MSE}=\underset{\left(i,j\right)\in P\cup N}{\sum }{\left({\hat{s}}_{i,j}-s_{i,j}\right)}^{2},$$

where *ŝ_i,__j_* = 1 if (*i*, *j*) ∈ *P*, *ŝ_i,__j_* = – 1 if (*i*, *j*) ∈ *N*, and *s_i,__j_* stands for the potential score of gene pair (*i*, *j*) predicted by EXP2SL.

The second part of the objective function is inspired by the semi-supervised Bayesian personalized ranking (BPR) loss (Rendle et al., 2009), which uses the unknown labels to boost the prediction performance. In particular, the BPR loss is defined as

(5) $$L_{BPR}=\underset{\left(a,b\right)\in P,\left(c,d\right)\in U}{\sum }\log\;\sigma \left(s_{a,b}-s_{c,d}\right)+\underset{\left(c,d\right)\in U,\left(e,f\right)\in N}{\sum }\log\;\sigma \left(s_{c,d}-s_{e,f}\right),$$

where *σ* stands for the sigmoid function $\sigma (x)=\frac{1}{1+e^{-x}}$. This objective function aims to enlarge the margins of the predicted scores between positive SL and unknown pairs, as well as those between the unknown and negative SL pairs. To calculate this loss, we sample the negative and unknown pairs with the sample number equal to the positive pairs during model training.

The above MSE and BPR objective functions are further combined with an L2 regularizier over all the learnable model parameters to construct the final objective function of our EXP2SL model, that is,

(6) $$L\left(\theta \right)=L_{MSE}+\lambda_{1}L_{BPR}+\lambda_{2}||\theta |{|}^{2},$$

where *θ* denotes the model parameters, and λ_1_ and λ_2_ stand for the weight parameters controlling the contributions of the BPR loss and the L2 regularization term, respectively.

To train the EXP2SL model, we used the Adam optimizer (Kingma and Ba, 2014) with the default learning rate 0.001 and the number of training epochs 1,000. We also clipped the gradient if it was larger than 5 to stabilize the training process. We implemented our model with PyTorch 1.0.1 (Paszke et al., 2017).

#### Hyper-Parameters

The hyper-parameters of our model include the weight of the BPR loss λ_1_ from [16, 32, 64, 128], the weight of the L2 regularization λ_2_ from [0.1, 0.05, 0.01, 0.005, 0.0001], the number of encoding layers from [0, 1, 2, 3, 4], and the dimension of hidden features *d* from [32, 64, 128, 256]. For each cell line, a grid search was performed to select the best combination of hyper-parameter settings from the above mentioned ranges, according to the AUC scores achieved by five repeats of 5-fold cross validations under the “split pair” setting (*i.e.*, gene pairs were randomly split into training and test sets). Details about the cross-validation settings can be found in *Performance Evaluation*. The baseline models were tuned using the same strategy, and the ranges for hyper-parameters in each baseline model are described in the *Baseline Models*.

#### Extraction of Feature Importance

Here, we used the saliency map-based approach proposed in (Simonyan et al., 2013) to evaluate the importance of each position along the 978-dimensional input features ${\left\{f_{i}\right\}}_{i=1}^{N}$. The basic idea of this method is to calculate the gradients of the output score with respect the to the input features, and the larger absolute values of gradients would suggest the more importance of the corresponding feature dimension. After the training process, the positive and negative SL pairs of each cell line are fed into the EXP2SL model, and the corresponding importance for each input feature dimension is calculated by

(7) $$w=\underset{\left(i,j\right)\in P\cup N}{\sum }|\frac{\partial s_{i,j}}{\partial f_{i}}\left|+\right|\frac{\partial s_{i,j}}{\partial f_{j}}|,$$

where *s_i,__j_* is the predicted confidence score of gene pair (*i*, *j*), and ***w*** is a 978-dimensional vector containing the importance score of each dimension of the input L1000 gene expression profiles. To reduce the variance caused by random initialization of network parameters and random sampling of the unknown and negative gene pairs for calculating the BPR loss during the training process, we also take the summation of ***w*** vectors from 10 trained EXP2SL models to obtain the final importance scores for the 978 feature dimensions. The top 50 ranked features are then selected for each cell line. We examined the overlaps of the selected features between cell lines and calculated the over-representations of functional gene sets and pathways using the WebGestalt server (Liao et al., 2019).

### Baseline Models

#### Logistic Regression

We used the logistic regression (LR) model implemented based on scikit-learn (Buitinck et al., 2013). The L1000 expression profiles were used as input to the LR model. For each pair of input genes (*i*,*j*), the features of genes *i* and *j* (denoted as ***f*** *_i_* and ***f*** *_j_*, respectively) were concatenated before being fed into the LR model. Since LR may produce different results for pairs (*i*, *j*) and (*j*, *i*), each of the two pairs were treated as an individual input with the same label in the training phase. In the test phase, the prediction values from both inputs were then averaged to obtain the final prediction score. The inverse of regularization strength (a hyper-parameter) was chosen from [10, 1, 0.5, 0.1, 0.05, 0.01].

#### Random Forest

We used the random forest (RF) classifier implemented based on scikit-learn (Buitinck et al., 2013). The input and output of RF were the same as those of LR described above. The number of trees was selected from [32, 64, 128] and the maximum depth of the trees was selected from [8, 16, None], where “None” means that the trees will keep expanding until no node can be split.

#### Support Vector Machine

We used the support vector machine (SVM) classifier implemented based on scikit-learn (Buitinck et al., 2013). The input and output of SVM were the same as those of LR and RF described above. The only hyper-parameter, the inverse of regularization strength, was selected from [100, 50, 10, 5, 1, 0.5, 0.1].

#### Gradient Boosting Decision Tree

We used the gradient-boosting decision tree (GBDT) classifier implemented by the XGBoost project (Chen and Guestrin, 2016). The input and output of GBDT were the same as other classifiers described above. The number of trees was selected from [32, 64, 128] and the maximum depth of the trees was selected from [4, 8, 16].

#### NetLapRLS

NetLapRLS (Xia et al., 2010) (a semi-supervised regressor) was implemented based on pyDTI (https://github.com/stephenliu0423/PyDTI). As NetLapRLS treats symmetric gene pairs (*i*, *j*) and (*j*, *i*) in the same way, there is no need to average the predictions of both pairs. Three types of similarity matrices were used as the input to NetLapRLS: 1) The protein-protein interaction (PPI) similarity matrix *S_p_*, *i.e.*, the pairwise PPI similarities between all pairwise genes used in the cell line. The human PPI data were obtained from the STRING database v11 (Szklarczyk et al., 2014). Protein pairs marked with STRING scores larger than 0.8 were considered positive interaction pairs in the PPI network. The PPI similarity between two proteins (*i*, *j*) were calculated as the Jaccard similarity of their interaction partners in the PPI network, that is,

(8) $$S_{p}\left(i,j\right)=\frac{\left|N\left(i\right)\cap N\left(j\right)\right|}{\left|N\left(i\right)\cup N\left(j\right)\right|},$$

where *N*(*x*) stands for the neighbors of protein *x* in the PPI network. 2) The L1000 profile similarity matrix *S_l_*, *i.e.*, the absolute values of the pairwise L1000 profile similarities between all the genes used in the cell line. The L1000 profile similarity between two genes were calculated as the Pearson correlation between their L1000 gene expression profiles. 3) The combination of both PPI and L1000 similarities, calculated as 1 – (1 – *S_p_*)(1 – *S_l_*). The best hyper-parameter settings were selected from all the combinations over γ*_d_* = γ*_t_* from [0.0001, 0.001, 0.01, 0.1, 1] and *β_d_* = *β_t_* from [0.003, 0.03, 0.3,3, 30].

## Results

### Cell-Line Specificity of SL Interactions

**Figure 2:**
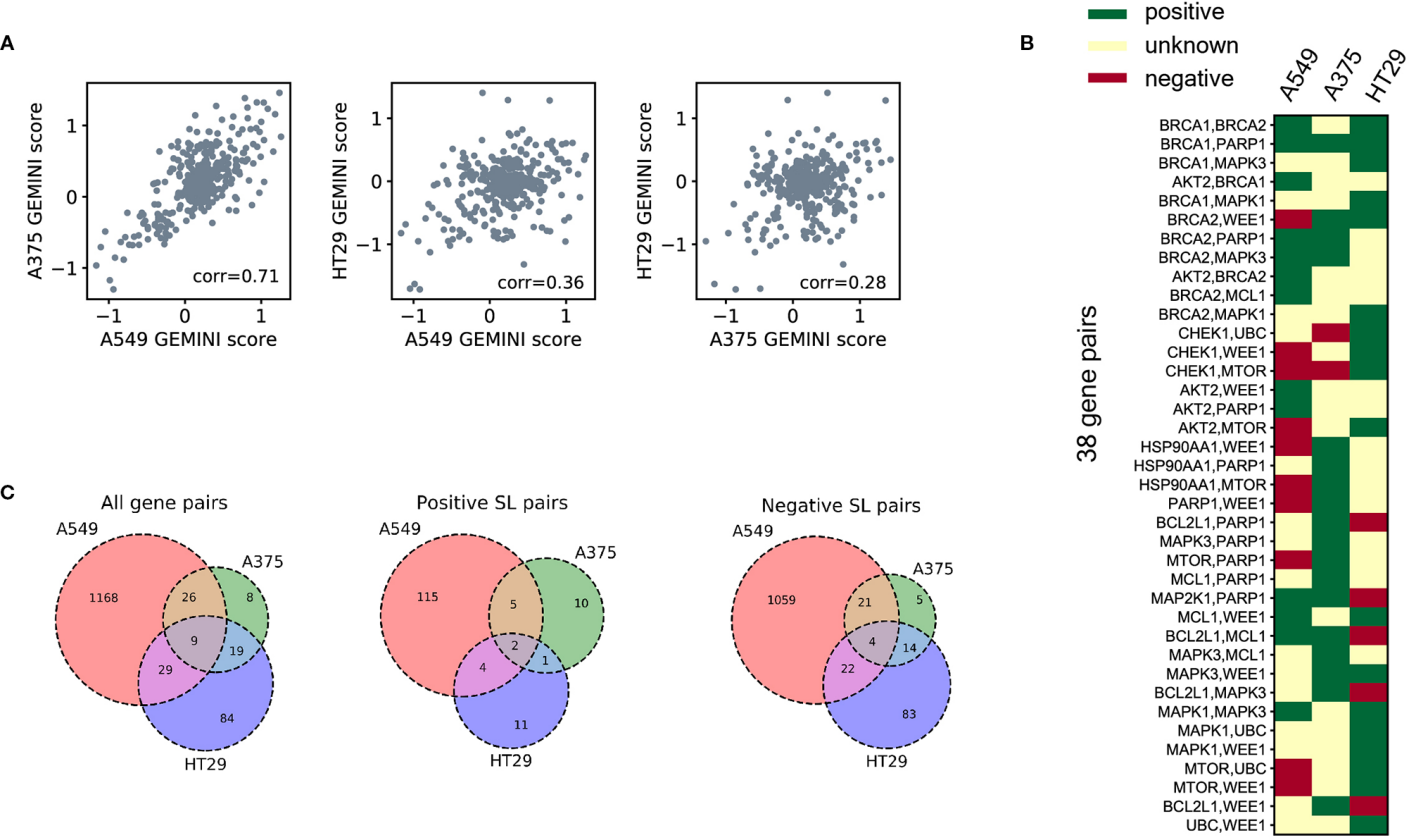
SL datasets for three human cell lines. **(A)** Correlations of the GEMINI scores between three different cell lines for the same gene pairs measured in the Big Papi dataset. **(B)** The binary SL labels for the gene pairs in the Big Papi dataset. The 38 gene pairs measured in all the three cell lines and with at least one positive SL label are included in the figure. **(C)** The Venn diagrams of all labeled SL pairs, positive SL pairs, and negative SL pairs used in our dataset, which were constructed from the Big Papi dataset and other available CRISPR-Cas9 based experimental screens in the literature.

To demonstrate the cell-line specificity of SL interactions, we examined 378 CRISPR knockout pairs screened in different cell lines from the Big Papi SynLet library (Najm et al., 2018). Their SL scores were calculated by GEMINI (Zamanighomi et al., 2019), a computational tool for identifying SL interactions from pairwise CRISPR knockout screens. Three cell lines were used in our performance evaluation, including A549, A375, and HT29. Among these three cell lines, A549 and A375 exhibited relatively high correlation (Pearson correlation 0.71, **Figure 2A**) in GEMINI scores, which measure the strength of the SL interactions. Meanwhile, the correlations between HT29 and the other two cell lines are relatively low (Pearson correlations 0.36 and 0.28, **Figure 2A**). These results indicate that the SL interaction patterns between the same gene pairs in different cell lines can be quite different.

Next, we examined the positive and negative SL samples selected from the Big Papi dataset according to the criteria described in *SL Labels*. By comparing the SL labels of the same gene pairs in the three cell lines, we found that most gene pairs have inconsistent labels cross different cell lines (**Figure 2B**). There are 38 gene pairs with at least one positive label in the three cell lines, but only one of them (*i.e.*, the *BRCA1*-*PARP1* gene pair) is always labeled as a positive SL. Among these 38 gene pairs, 16 have negative labels in one cell line but positive labels in another one.

Based on the above observation that most SL pairs were not conserved across different cell lines, we built prediction models for each cell line separately. In addition to the Big Papi dataset, we also included the data from other literature (Shen et al., 2017; Zhao et al., 2018), which further enlarged the SL data of cell line A549. The overlaps of gene pairs used as labeled training samples between the three cell lines are shown in **Figure 2C**.

### Performance Evaluation

We compared the performance of our model to that of several baseline methods through cross-validation on the aforementioned datasets for the three cell lines. LR, RF, SVM, and GBDT were selected as the baseline methods because they are the machine learning baseline models and accept vector input, which is suitable for our case. NetLapRLS is also used as a baseline model, as it is a well-established semi-supervised method that accepts network input and which can be used to test the effectiveness of other features, such as the PPI network. Two settings were used to split the training and test samples. The first one was called “split pair” in which gene pairs were randomly split into training and test sets. The second one was called “split gene” in which, for each test gene pair, at least one gene is not seen in training data. The “split gene” setting was mainly used to test whether the prediction can be generalized to unseen genes, which is more challenging. Note that the splitting was performed over positive and negative SL pairs, and our model also utilized the unknown pairs during the training process.

Area under the receiver operating characteristic curve (AUC), area under the precision-recall curve (AUPR), F1 score, accuracy, precision, sensitivity and selectivity were used to evaluate the classification performance (**Tables 2** and **3**). The receiver operating characteristic (ROC) and precision-recall (PR) curves achieved by EXP2SL and the baseline models are shown in **Figures S2–S3**. Under the “split pair” setting, all the models achieved relatively high performance, which indicates that the prediction problem defined under this setting was relatively easy. The performance of our model was comparable with the top-performing baseline methods under this setting. However, under the more practical “split gene” setting in which we wished to predict SL pairs containing novel genes without experimental screen data (due to the limited existing experimental data), the SL prediction task became difficult as all the models achieved relatively lower AUC and AUPR scores than those under the “split pair” setting. However, our model exhibited a significantly better performance than that of all the baseline models under this “split gene” setting. EXP2SL achieved the best performance in at least 6/7 metrics for all the three cell lines (**Table 3**). We also tested our model and the baseline methods with a less strict threshold for defining the positive SL pairs (*i.e.*, 10%), and our model also achieved a better performance than that of the baseline methods (**Tables S1–S2**).

**Table 2:** Performance evaluation in three different cell lines under the “split pair” setting. The mean and standard deviation (in brackets) of metrics over 10 repeats of 5-fold cross-validations are shown. The best results for each cell line and each metric are marked in bold.

Dataset	Model name	AUC	AUPR	F1	Accuracy	Precision	Sensitivity	Specificity
A549	LR	0.863 (0.041)	0.556 (0.089)	0.577 (0.068)	0.913 (0.030)	0.622 (0.109)	0.573 (0.033)	0.952 (0.032)
	RF	0.854 (0.039)	0.552 (0.076)	0.567 (0.069)	0.912 (0.027)	0.600 (0.104)	0.559 (0.032)	0.952 (0.026)
	SVM	0.809 (0.038)	0.505 (0.084)	0.555 (0.060)	0.914 (0.019)	0.610 (0.104)	0.523 (0.037)	**0.958 (0.019)**
	GBDT	0.847 (0.039)	0.520 (0.086)	0.552 (0.065)	0.908 (0.029)	0.573 (0.120)	0.552 (0.037)	0.948 (0.033)
	NetLapRLS(L1000)^1^	0.760 (0.044)	0.344 (0.088)	0.407 (0.068)	0.845 (0.034)	0.357 (0.119)	0.512 (0.039)	0.883 (0.038)
	NetLapRLS(PPI) ^2^	0.760 (0.045)	0.344 (0.090)	0.407 (0.079)	0.845 (0.034)	0.357 (0.130)	0.512 (0.032)	0.883 (0.037)
	NetLapRLS(combined) ^3^	0.827 (0.042)	0.488 (0.091)	0.519 (0.061)	0.898 (0.025)	0.523 (0.100)	0.539 (0.017)	0.938 (0.027)
	EXP2SL(no BPR loss) ^4^	0.866 (0.038)	**0.576 (0.086)**	**0.583 (0.071)**	**0.916 (0.032)**	**0.638 (0.135)**	0.565 (0.036)	0.955 (0.035)
	EXP2SL(PPI) ^5^	0.870 (0.041)	0.574 (0.078)	0.583 (0.055)	0.915 (0.020)	0.636 (0.081)	0.573 (0.039)	0.954 (0.020)
	EXP2SL	**0.871 (0.044)**	0.573 (0.083)	0.582 (0.070)	0.914 (0.024)	0.634 (0.084)	**0.579 (0.063)**	0.952 (0.023)
A375	LR	0.994 (0.004)	0.983 (0.006)	0.981 (0.011)	0.989 (0.007)	0.967 (0.018)	1.000 (0.015)	0.984 (0.011)
	RF	0.997 (0.004)	0.990 (0.015)	0.987 (0.016)	0.993 (0.007)	0.977 (0.028)	1.000 (0.010)	0.990 (0.010)
	SVM	0.991 (0.004)	0.978 (0.017)	0.972 (0.020)	0.984 (0.008)	0.962 (0.033)	0.991 (0.000)	0.983 (0.009)
	GBDT	0.999 (0.009)	0.997 (0.013)	0.993 (0.019)	0.996 (0.013)	0.993 (0.020)	0.994 (0.022)	0.997 (0.012)
	NetLapRLS(L1000) ^1^	0.989 (0.005)	0.983 (0.006)	0.969 (0.014)	0.976 (0.013)	0.956 (0.026)	0.990 (0.012)	0.966 (0.022)
	NetLapRLS(PPI) ^2^	0.990 (0.002)	0.985 (0.003)	0.972 (0.012)	0.978 (0.010)	0.956 (0.021)	0.995 (0.000)	0.966 (0.017)
	NetLapRLS(combined) ^3^	0.994 (0.007)	0.990 (0.007)	0.983 (0.016)	0.987 (0.018)	0.971 (0.026)	1.000 (0.000)	0.979 (0.033)
	EXP2SL(no BPR loss) ^4^	1.000 (0.003)	1.000 (0.011)	1.000 (0.013)	1.000 (0.008)	1.000 (0.023)	1.000 (0.000)	1.000 (0.012)
	EXP2SL(PPI)^5^	1.000 (0.008)	1.000 (0.010)	1.000 (0.015)	1.000 (0.014)	1.000 (0.026)	1.000 (0.000)	1.000 (0.023)
	EXP2SL	**1.000 (0.012)**	**1.000 (0.029)**	**1.000 (0.026)**	**1.000 (0.016)**	**1.000 (0.043)**	**1.000 (0.000)**	**1.000 (0.021)**
HT29	LR	0.967 (0.015)	0.861 (0.049)	0.851 (0.032)	0.958 (0.012)	0.855 (0.053)	0.895 (0.048)	0.968 (0.017)
	RF	0.955 (0.020)	0.821 (0.067)	0.824 (0.030)	0.947 (0.005)	0.792 (0.039)	0.899 (0.073)	0.955 (0.005)
	SVM	0.949 (0.017)	0.765 (0.079)	0.808 (0.065)	0.943 (0.015)	0.744 (0.069)	**0.942 (0.100)**	0.941 (0.018)
	GBDT	**0.973 (0.016)**	0.880 (0.061)	0.855 (0.029)	**0.960 (0.015)**	0.861 (0.065)	0.897 (0.040)	**0.969 (0.021)**
	NetLapRLS(L1000) ^1^	0.935 (0.017)	0.738 (0.094)	0.778 (0.064)	0.941 (0.025)	0.786 (0.139)	0.836 (0.053)	0.954 (0.034)
	NetLapRLS(PPI) ^2^	0.927 (0.024)	0.729 (0.086)	0.772 (0.053)	0.939 (0.008)	0.787 (0.048)	0.822 (0.056)	0.953 (0.009)
	NetLapRLS(combined) ^3^	0.939 (0.019)	0.764 (0.094)	0.784 (0.054)	0.939 (0.020)	0.778 (0.107)	0.850 (0.035)	0.949 (0.026)
	EXP2SL(no BPR loss^4^	0.957 (0.026)	0.834 (0.071)	0.826 (0.043)	0.943 (0.017)	0.779 (0.088)	0.926 (0.051)	0.946 (0.023)
	EXP2SL(PPI)^5^	0.967 (0.018)	0.869 (0.033)	0.851 (0.026)	0.956 (0.011)	0.838 (0.067)	0.912 (0.084)	0.962 (0.022)
	EXP2SL	0.969 (0.008)	**0.880 (0.027)**	**0.866 (0.027)**	0.959 (0.012)	**0.872 (0.055)**	0.903 (0.049)	0.968 (0.018)

^1^The NetLapRLS method using only the L1000 similarity.

^2^The NetLapRLS method using only the PPI similarity.

^3^The NetLapRLS method using the combination of L1000 and PPI similarities.

^4^The EXP2SL model without the BPR loss.

^5^The EXP2SL model with additional PPI information incorporated by a graph convolution module.

**Table 3:** Performance evaluation in three different cell lines under the “split gene” setting. The mean and standard deviation (in brackets) of metrics over 10 repeats of 5-fold cross-validations are shown. The best results for each cell line and each metric are marked in bold.

Dataset	Model name	AUC	AUPR	F1	Accuracy	Precision	Sensitivity	Specificity
A549	LR	0.709 (0.039)	0.328 (0.050)	0.373 (0.039)	0.816 (0.044)	0.404 (0.070)	0.435 (0.059)	0.853 (0.058)
	RF	0.715 (0.037)	0.348 (0.052)	0.379 (0.038)	0.850 (0.024)	0.461 (0.058)	0.394 (0.038)	0.896 (0.027)
	SVM	0.708 (0.026)	0.340 (0.051)	0.380 (0.032)	0.838 (0.020)	0.433 (0.037)	0.432 (0.060)	0.876 (0.030)
	GBDT	0.715 (0.030)	0.333 (0.051)	0.363 (0.032)	0.841 (0.043)	0.401 (0.094)	0.399 (0.057)	0.888 (0.054)
	NetLapRLS(L1000) ^1^	0.668 (0.024)	0.252 (0.038)	0.321 (0.021)	0.815 (0.016)	0.294 (0.057)	0.407 (0.029)	0.858 (0.018)
	NetLapRLS(PPI) ^2^	0.668 (0.030)	0.252 (0.048)	0.321 (0.041)	0.815 (0.016)	0.294 (0.070)	0.407 (0.036)	0.858 (0.019)
	NetLapRLS(combined) ^3^	0.685 (0.032)	0.331 (0.043)	0.371 (0.035)	0.863 (0.021)	0.426 (0.083)	0.368 (0.046)	**0.918 (0.027)**
	EXP2SL(no BPR loss) ^4^	0.699 (0.032)	0.358 (0.053)	0.389 (0.035)	0.857 (0.033)	0.450 (0.083)	0.401 (0.043)	0.906 (0.042)
	EXP2SL(PPI) ^5^	0.755 (0.024)	0.390 (0.044)	0.419 (0.034)	0.861 (0.041)	**0.465 (0.079)**	**0.450 (0.047)**	0.903 (0.054)
	EXP2SL	**0.756 (0.030)**	**0.392 (0.043)**	**0.419 (0.024)**	**0.863 (0.048)**	0.458 (0.073)	0.448 (0.050)	0.907 (0.061)
A375	LR	0.945 (0.026)	0.884 (0.050)	0.874 (0.046)	0.930 (0.034)	0.866 (0.054)	0.897 (0.031)	0.925 (0.033)
	RF	0.947 (0.028)	0.886 (0.045)	0.891 (0.038)	0.934 (0.032)	0.865 (0.039)	0.938 (0.025)	0.917 (0.027)
	SVM	0.924 (0.027)	0.860 (0.047)	0.873 (0.035)	0.916 (0.026)	0.864 (0.044)	0.915 (0.032)	0.905 (0.030)
	GBDT	0.923 (0.019)	0.852 (0.056)	0.875 (0.048)	0.920 (0.022)	0.862 (0.047)	0.926 (0.040)	0.909 (0.047)
	NetLapRLS(L1000) ^1^	0.915 (0.050)	0.822 (0.054)	0.821 (0.085)	0.895 (0.052)	0.827 (0.020)	0.889 (0.112)	0.933 (0.069)
	NetLapRLS(PPI) ^2^	0.915 (0.033)	0.823 (0.063)	0.821 (0.046)	0.895 (0.036)	0.827 (0.047)	0.889 (0.029)	0.933 (0.025)
	NetLapRLS(combined) ^3^	0.921 (0.022)	0.837 (0.054)	0.840 (0.045)	0.912 (0.030)	0.858 (0.063)	0.869 (0.024)	0.955 (0.025)
	EXP2SL(no BPR loss) ^4^	0.952 (0.035)	0.895 (0.052)	0.905 (0.042)	0.943 (0.031)	0.873 (0.045)	**0.967 (0.032)**	0.922 (0.033)
	EXP2SL(PPI) ^5^	**0.976 (0.028)**	**0.936 (0.028)**	**0.932 (0.022)**	**0.966 (0.024)**	**0.919 (0.046)**	0.959 (0.062)	**0.961 (0.055)**
	EXP2SL	0.976 (0.023)	0.935 (0.055)	0.926 (0.046)	0.964 (0.030)	0.902 (0.045)	0.965 (0.038)	0.960 (0.025)
HT29	LR	0.754 (0.056)	0.417 (0.075)	0.531 (0.041)	0.823 (0.050)	0.505 (0.059)	0.709 (0.048)	0.841 (0.067)
	RF	0.846 (0.030)	0.494 (0.062)	0.587 (0.037)	0.858 (0.028)	0.524 (0.057)	0.763 (0.057)	0.869 (0.026)
	SVM	0.827 (0.034)	0.465 (0.044)	0.595 (0.043)	0.857 (0.032)	0.539 (0.066)	**0.792 (0.056)**	0.863 (0.036)
	GBDT	0.823 (0.057)	0.452 (0.071)	0.546 (0.044)	0.822 (0.046)	0.495 (0.055)	0.758 (0.026)	0.839 (0.057)
	NetLapRLS(L1000) ^1^	0.801 (0.043)	0.441 (0.056)	0.542 (0.042)	0.826 (0.042)	0.475 (0.079)	0.755 (0.070)	0.837 (0.055)
	NetLapRLS(PPI) ^2^	0.794 (0.026)	0.423 (0.047)	0.525 (0.030)	0.818 (0.022)	0.458 (0.069)	0.761 (0.040)	0.828 (0.034)
	NetLapRLS(combined) ^3^	0.814 (0.029)	0.464 (0.081)	0.550 (0.045)	0.840 (0.043)	0.479 (0.062)	0.758 (0.073)	0.853 (0.055)
	EXP2SL(no BPR loss) ^4^	0.788 (0.035)	0.481 (0.040)	0.577 (0.059)	0.830 (0.037)	0.531 (0.086)	0.752 (0.040)	0.835 (0.048)
	EXP2SL(PPI) ^5^	0.865 (0.032)	0.553 (0.038)	0.612 (0.024)	0.872 (0.012)	0.563 (0.049)	0.766 (0.046)	0.882 (0.018)
	EXP2SL	**0.866 (0.039)**	**0.558 (0.066)**	**0.620 (0.046)**	**0.877 (0.028)**	**0.577 (0.065)**	0.756 (0.065)	**0.890 (0.035)**

^1^The NetLapRLS method using only the L1000 similarity.

^2^The NetLapRLS method using only the PPI similarity.

^3^The NetLapRLS method using the combination of L1000 and PPI similarities.

^4^The EXP2SL model without the BPR loss.

^5^The EXP2SL model with additional PPI information incorporated by a graph convolution module.

### Ablation Study and Feature Comparison

To evaluate the contribution of the semi-supervised objective function to the final prediction, we tested our EXP2SL model without the BPR loss. That is, we modified the objective function in Equation 6 and used only the MSE loss and the L2 regularization term; our model can thus be trained in a supervised manner. An obvious decrease in performance under the “split gene” setting could be observed when we removed the BPR loss (see the “EXP2SL(no BPR loss)” row in **Table 3**). Therefore, the results demonstrated that the semi-supervised objective function had an important contribution to the prediction performance of our model.

One of the baseline models, NetLapRLS, can also incorporate different similarity matrices (*i.e.*, the L1000 profile similarities, the PPI similarities, and the combined similarities, as described in *NetLapRLS*), thus allowing the comparison between different settings using different input information. The NetLapRLS models with L1000 profile similarities and with PPI similarities as the input features achieved similar performance, and the combination of both features only led to a slight increase in performance in most cases. In general, the performance of NetLapRLS was worse than EXP2SL.

We also incorporated the PPI network into our EXP2SL framework (denoted as EXP2SL (PPI) in **Tables 2** and **3**) using a graph convolution network (Lei et al., 2017), as described in **Supporting Material** and **Figure S1**. In this case, no significant improvement in AUC and AUPR scores was observed after adding the PPI network information (*p* values larger than 0.1 for all the cell lines in both conditions, Wilcoxon rank-sum test). These results indicate that using only the L1000 gene expression profiles is adequate to enable the models to capture useful features for accurately predicting SL interactions.

### Feature Importance Analysis

We used the scheme described in *Extraction of Feature Importance* to extract the important features based on the saliency map approach (Simonyan et al., 2013). Those features (*i.e.*, the corresponding expression levels of 978 genes) ranked among the top 50 (about 5% from the 978-dimensional features) were selected as the important features for each cell line. Among the selected feature sets, there is only one gene shared across all the three cell lines, that is, *AKT1*. AKT1 is known as a serine/threonine protein kinase, which regulates many viability related cellular processes, including proliferation, apoptosis, and cell survival (Chen et al., 2001; Lee et al., 2011). Most features were considered as the top 50 important features only in one cell line (47, 46, and 46 unique important features for A549, A375, and HT29, respectively), which suggests that the prediction may rely on the specific gene expression landscapes in different cell lines.

We also checked the over-representation of functional gene sets and pathways among the selected important features of the three cell lines using the WebGestalt server (Liao et al., 2019). The gene ontology (GO) related to biological processes was first used to examine the enriched functional annotations of the selected feature sets (**Tables S3–S5**). The enriched GO terms were ranked according to the false discovery rate (FDR) scores and *p* values. As a result, the top 10 enriched functional annotations for the selected features of HT29 contains the regulation of cell death, proliferation, and apoptosis (*p* values < 10^–^^6^ and FDRs < 10^–^^3^), which are cell viability related functions. Then, we also checked the over-representation of selected genes among the KEGG pathways using the WebGestalt server (Liao et al., 2019) (**Tables S6–S8**). Among the top 10 enriched pathways ranked according to the FDR scores and *p* values, we found multiple cancer-related pathways for cell line HT29 and also cell cycle or cancer-regulatory pathways for A375 and A549, *e.g.*, the *p53* and *ERBB* signaling pathways. All these results indicated that the selected features are probably related to the regulation of cell viability.

## Conclusion

In this paper, we proposed a semi-supervised neural network based method, EXP2SL, to accurately predict cell-line specific SL interactions. Our method exploits the L1000 expression profiles measured from the shRNA knockdown experiments performed in different cell lines to learn the cell-line specific SL interactions from the labeled data generated by CRISPR-Cas9 double-knockout based screens. In addition, a semi-supervised objective function is designed to make use of the large amount of unlabeled data. Tests on three datasets corresponding to three different cell lines showed that our model achieved better performance than the baseline models. At the same time, we verified that the L1000 gene expression profiles and the semi-supervised objective function are useful in SL prediction. Moreover, we analyzed the most important genes among the whole L1000 gene expression profiles, and found that the top attributing genes are related to the regulation of cell viability, which suggested that our model may pay more attention to such meaningful components of the whole gene expression profiles.

The major contributions of our work are the demonstration of L1000 expression profiles as effective features for SL prediction, and a novel semi-supervised neural network algorithm to accurately capture SL interactions. To our best knowledge, our model is the *first* computational approach for predicting cell-line specific synthetic lethal interactions, which may potentially benefit the target identification for specific tissue or cancer types. However, the application of our model may be limited in certain cancer types with high heterogeneity. Another limitation of our model is the dependence of the available L1000 gene expression profiles as input to EXP2SL. Although the L1000 expression profiles of more than 3,500 genes have been measured by shRNA knockdown experiments in the three cell lines analyzed in this work, there exist some cell lines with a paucity of data, which may thus limit the applications of our model on such cell lines.

## Data Availability Statement

The datasets analyzed for this study can be found in the L1000 datasets GSE92742 (https://www.ncbi.nlm.nih.gov/geo/query/acc.cgi?acc=GSE92742) and the GEMINI datasets (Additional file 2 in https://genomebiology.biomedcentral.com/articles/10.1186/s13059-019-1745-9#additional-information). Codes and processed data for this study can be found in https://github.com/FangpingWan/EXP2SL.

## Author Contributions

JZ, DZ, and FW conceived the project. FW, SL, and TT designed the method. FW, SL, YL, and DZ performed the analyses. All the authors contributed to the writing of the manuscript.

## Funding

This work was supported in part by the National Natural Science Foundation of China [61872216, 81630103, 31900862]. The authors declare that this study received funding from the Turing AI Institute of Nanjing and the Zhongguancun Haihua Institute for Frontier Information Technology. The funders were not involved in the study design, collection, analysis, interpretation of data, the writing of this article or the decision to submit it for publication.

## Conflict of Interest

YL was employed by company Silexon AI Technology Co. Ltd.

The remaining authors declare that the research was conducted in the absence of any commercial or financial relationships that could be construed as a potential conflict of interest.
