# Supplementary material for: EXP2SL: A Machine Learning Framework for Cell-Line-Specific Synthetic Lethality Prediction
Source: Front Pharmacol. 2020 Feb 28;11:112. doi: 10.3389/fphar.2020.00112 (PMC7058988; doi:10.3389/fphar.2020.00112)
Supplement: Supplementary file 1 [file DataSheet_1.pdf]

Supplementary Material for

EXP2SL: a Machine Learning Framework for Cell-Line Specific Synthetic Lethality Prediction

1 SUPPLEMENTARY METHOD

1.1 The implementation of EXP2SL with the PPI network incorporated by a graph convolution module

We use the same notations as in the main text, that is, the indices $1, 2, ..., N$ stand for the N genes with shRNA data from the LINCS L1000 project (Subramanian et al., 2017) in a given cell line. The L1000 gene expression profiles are denoted by $\{\boldsymbol{f}_i \in \mathbb{R}^{978}\}_{i=1}^{N}$. In addition, a corresponding PPI matrix $\boldsymbol{P} \in \mathbb{R}^{N \times N}$ is used to represent the connectivities between these genes. Here, $P_{i,j} = 1$ denotes the existence of an interaction between genes i and j, and $P_{i,j} = 0$ otherwise. The PPI network information is obtained from the STRING database (Szklarczyk et al., 2014), the same as in NetLapRLS as described in the main text.

For a given cell line, our model takes both gene L1000 expression profiles $\{\boldsymbol{f}_i\}_{i=1}^{N}$ and the PPI network $\boldsymbol{P}$ as inputs and processes them through three steps to predict the potential SL pairs between genes (Figure S1).

Step 1: a low-dimensional encoder. The gene features (*i.e.*, the L1000 expression profiles) are first transformed into the low-dimensional hidden representations $\{\boldsymbol{h}_i^0 \in \mathbb{R}^d\}_{i=1}^{N}$ through a single layer neural network, that is,

$$\boldsymbol{h}_i^0 = ReLU(\boldsymbol{W}_{encoder}\boldsymbol{f}_i + \boldsymbol{b}_{encoder}), \tag{S1}$$

where $ReLU(x)$ stands for the rectifier linear activation function $ReLU(x) = max(0, x)$, $\boldsymbol{W}_{encoder} \in \mathbb{R}^{d \times 978}$ and $\boldsymbol{b}_{encoder} \in \mathbb{R}^d$ ($d < 978$) denote the learnable parameters and $i \in \{1, 2, ..., N\}$.

Step 2: a graph convolution network. Next, a graph convolution network is adopted to enable information passing through the PPI network to update the features for each gene. Suppose that the number of graph convolution iterations is G. At the beginning of graph convolution, the inital representations of genes are $\{\boldsymbol{h}_i^0\}_{i=1}^{N}$. At g-th iteration ($g = 1, 2, ..., G$), the neighborhood information $\boldsymbol{m}_i^g$ passed to gene i through the PPI network is defined as:

$$\boldsymbol{m}_i^g = \alpha_i \sum_{j=1}^{N} P_{i,j} \cdot ReLU(\boldsymbol{W}_{ppi}^g \boldsymbol{h}_i^{g-1} + \boldsymbol{b}_{ppi}^g), \tag{S2}$$

where $\alpha_i = \frac{1}{\sum_{j=1}^{N} P_{i,j}}$ stands for the PPI normalization term, and $\boldsymbol{W}_{ppi}^g \in \mathbb{R}^{d \times d}$ and $\boldsymbol{b}_{ppi}^g \in \mathbb{R}^d$ are the learnable parameters.

Then, the passed information $\boldsymbol{m}_i^g$ is used to update the gene features through a gated recurrent unit (GRU) (Lei et al., 2018), that is,

$$\boldsymbol{h}_i^g = GRU(\boldsymbol{m}_i^g, \boldsymbol{h}_i^{g-1}), \tag{S3}$$

where $GRU(\boldsymbol{x}, \boldsymbol{y})$ is defined as

$$GRU(\boldsymbol{x}, \boldsymbol{y}) = (1 - z)\boldsymbol{y} + z\hat{\boldsymbol{y}}, \tag{S4}$$

$$\hat{\boldsymbol{y}} = tanh(\boldsymbol{W}_x\boldsymbol{x} + \boldsymbol{b}_x + r(\boldsymbol{W}_y\boldsymbol{y} + \boldsymbol{b}_y)), \tag{S5}$$

$$z = \sigma(\boldsymbol{W}_z[\boldsymbol{x}, \boldsymbol{y}] + \boldsymbol{b}_z), \tag{S6}$$

$$r = \sigma(\boldsymbol{W}_r[\boldsymbol{x}, \boldsymbol{y}] + \boldsymbol{b}_r). \tag{S7}$$

Here, z and r stand for the update and reset gates, respectively, $\boldsymbol{W}_x$, $\boldsymbol{W}_y \in \mathbb{R}^{d\times d}$, $\boldsymbol{b}_x$, $\boldsymbol{b}_y$, $\boldsymbol{b}_z$, $\boldsymbol{b}_r \in \mathbb{R}^d$, $\boldsymbol{W}_z$, $\boldsymbol{W}_r \in \mathbb{R}^{d\times 2d}$ denote the learnable parameters, $tanh(\cdot)$ denotes the hyperbolic tangent activation function, $\sigma(x) = \frac{1}{1+e^{-x}}$ denotes the sigmoid activation funciton, and $[\cdot, \cdot]$ denotes the concatenation operation.

Step 3: a linear layer. After L iterations of graph convolution, the updated gene features $\{\boldsymbol{h}_i^L\}_{i=1}^N$ are then used to predict SL interactions, in the same way as described in the main text. That is, for a gene pair (i, j), $i, j = 1, 2, ..., N$ and $i \neq j$, the predicted confidence score is calculated by

$$s_{i,j} = \frac{1}{2}(\boldsymbol{W}_{out}[\boldsymbol{h}_i^L, \boldsymbol{h}_j^L] + \boldsymbol{W}_{out}[\boldsymbol{h}_j^L, \boldsymbol{h}_i^L]) + \boldsymbol{b}_{out}, \tag{S8}$$

where $\boldsymbol{W}_{out} \in \mathbb{R}^{1\times 2d}$ and $\boldsymbol{b}_{out} \in \mathbb{R}$ stand for the learnable parameters.

2 SUPPLEMENTARY TABLES AND FIGURES

2.1 Figures

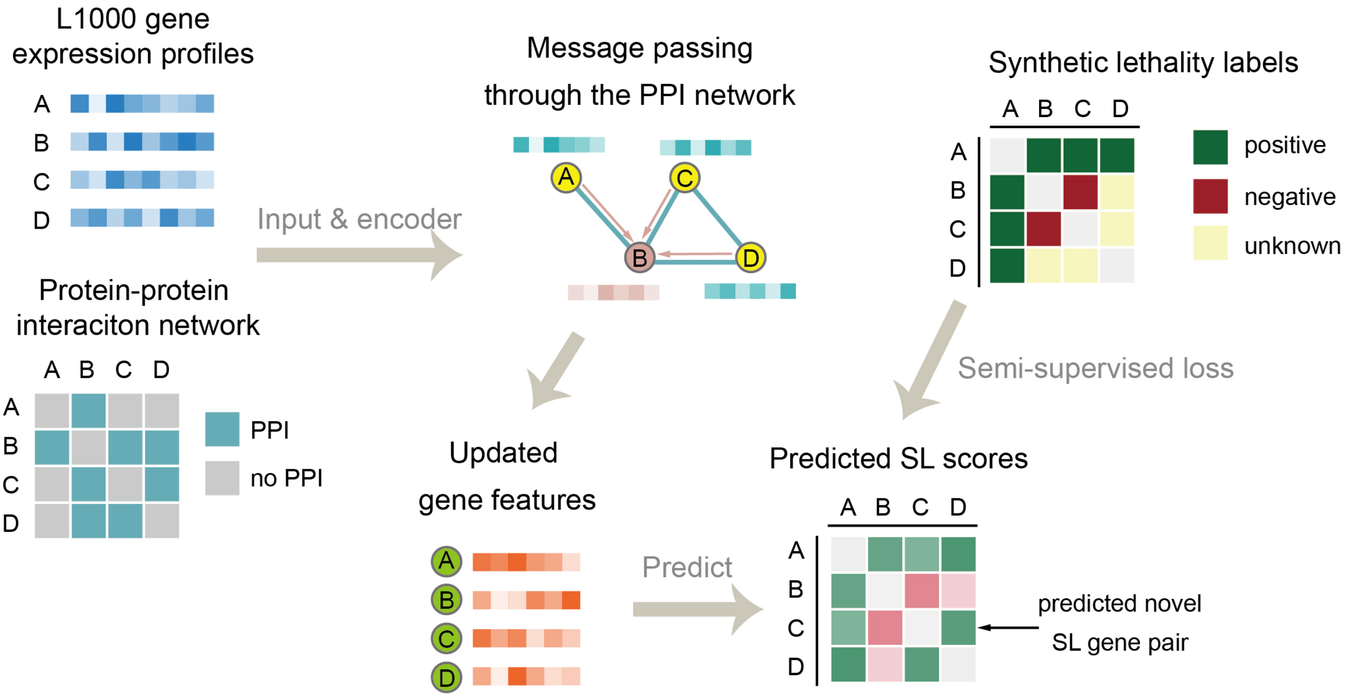

Figure S1. The work flow of EXP2SL method with the PPI incorporated by a graph convolution module.

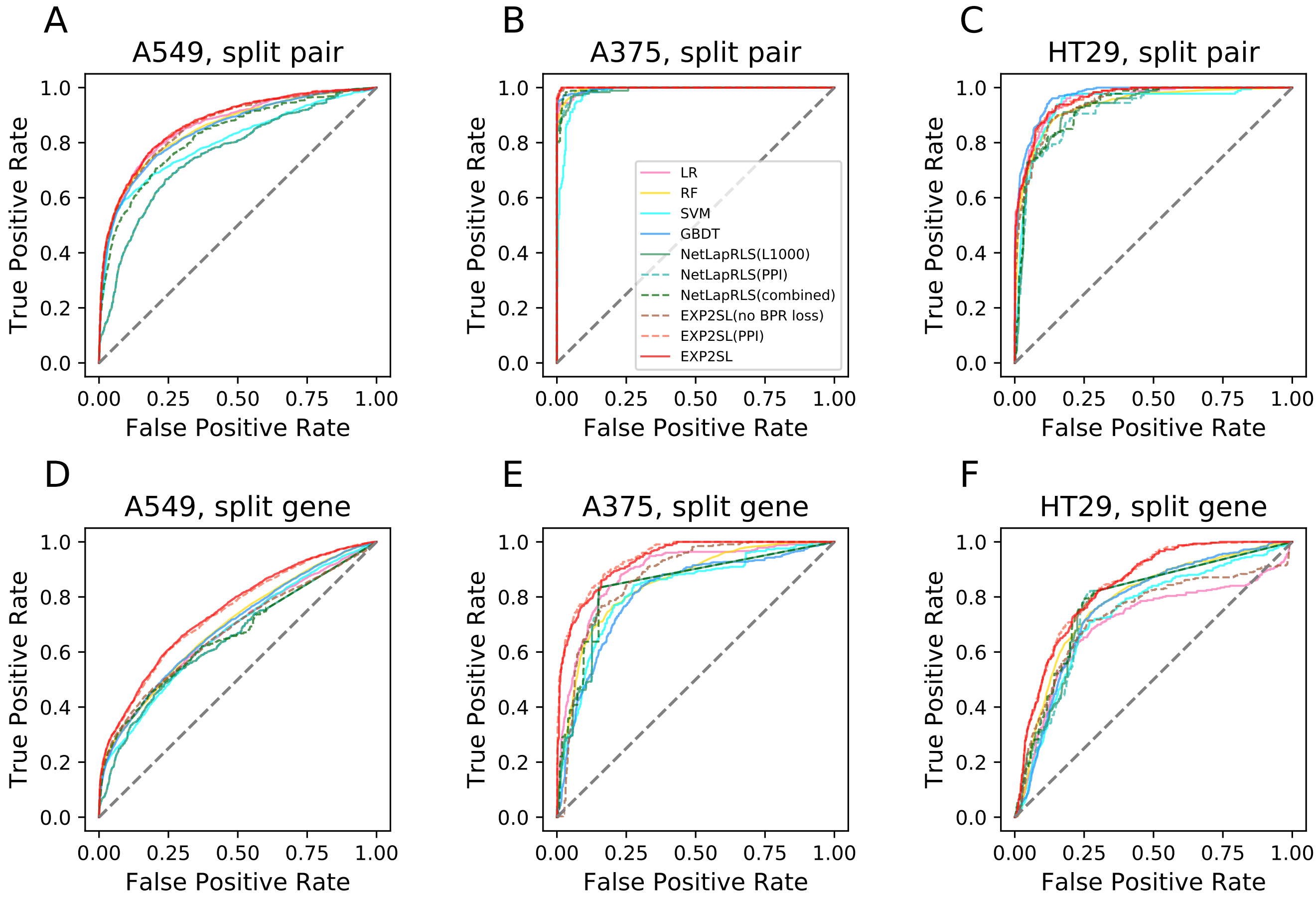

Figure S2. The receiver operator characteristic (ROC) curves achieved by EXP2SL and the baseline methods. Combined predictions over ten repeats of five cross-validations were evaluated for cell line A549 (A, D), A375 (B, E) and HT29 (C, F) under the "split gene" (A-C) and the "split pair" (D-F) settings are shown. The NetLapRLS models using only the L1000 similarities, only the PPI similarities and the combination of L1000 and PPI similarities are marked as "NetLapRLS(L1000)", "NetLapRLS(PPI)" and "NetLapRLS(combined)", respectively. The EXP2SL model without the BPR loss and with additional PPI information incorporated by a graph convolution module are marked as "EXP2SL(no BPR loss)" and "EXP2SL(PPI)", respectively.

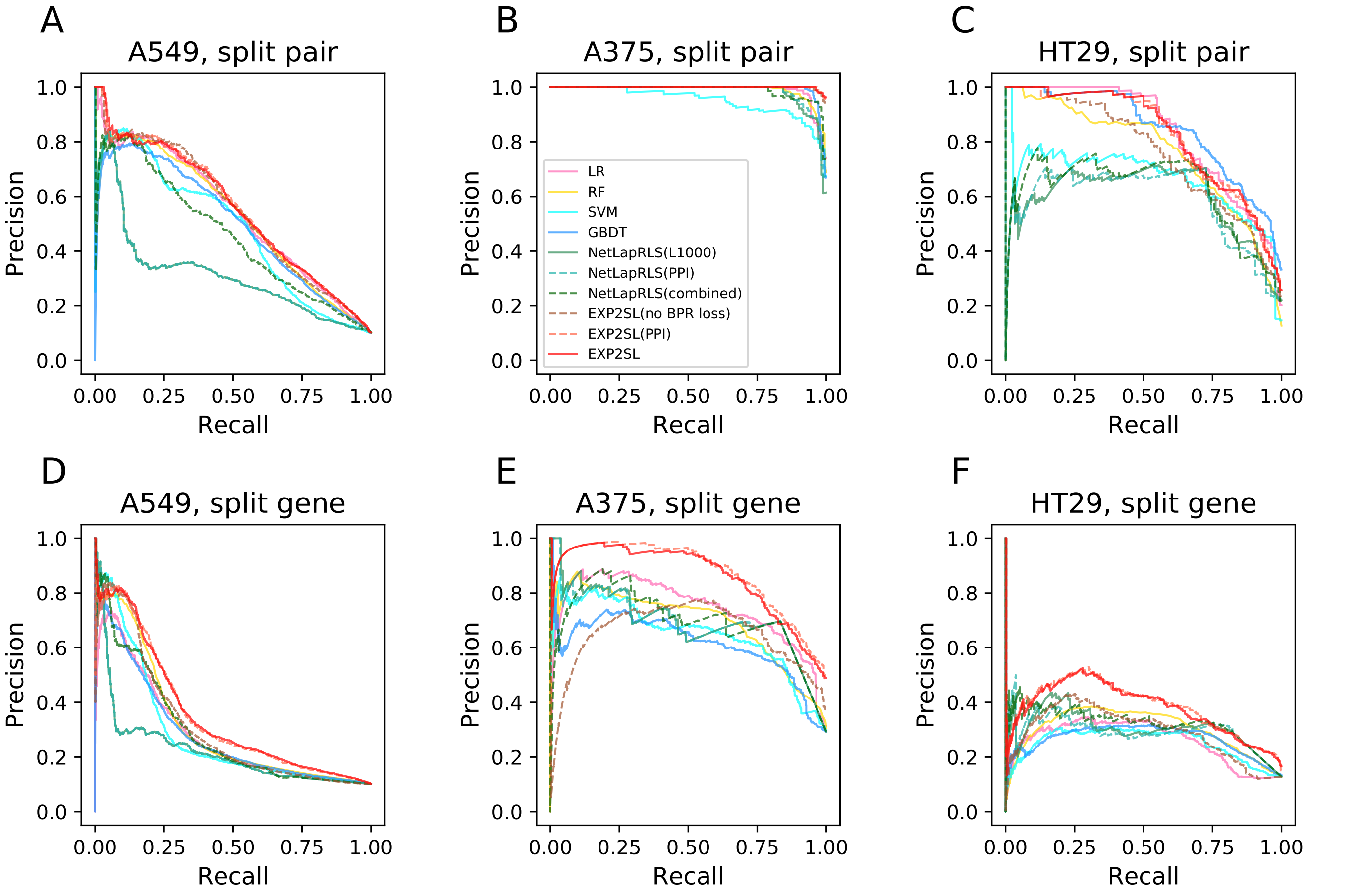

Figure S3. The precision-recall (PR) curves achieved by EXP2SL and the baseline methods. Combined predictions over ten repeats of five cross-validations were evaluated for cell line A549 (A, D), A375 (B, E) and HT29 (C, F) under the "split gene" (A-C) and the "split pair" (D-F) settings are shown. The NetLapRLS models using only the L1000 similarities, only the PPI similarities and the combination of L1000 and PPI similarities are marked as "NetLapRLS(L1000)", "NetLapRLS(PPI)" and "NetLapRLS(combined)", respectively. The EXP2SL model without the BPR loss and with additional PPI information incorporated by a graph convolution module are marked as "EXP2SL(no BPR loss)" and "EXP2SL(PPI)", respectively.

2.2 Tables

Table S1. Performance evaluation in three different cell lines under the "split pair" setting, with labels selected by threshold 10%. The mean and standard deviation (in brackets) of each metrics over 10 repeats are shown. The best results for each cell line and each metric are marked in bold.

Dataset	Model name	AUC	AUPR	F1	Accuracy	Precision	Sensitivity	Specificity
A549	LR	0.849 (0.041)	0.646 (0.075)	0.625 (0.056)	0.852 (0.047)	0.615 (0.096)	0.648 (0.047)	0.900 (0.063)
	RF	0.843 (0.040)	0.631 (0.072)	0.622 (0.051)	0.853 (0.038)	0.628 (0.078)	0.632 (0.040)	0.905 (0.049)
	SVM	0.799 (0.041)	0.567 (0.072)	0.575 (0.054)	0.846 (0.041)	0.620 (0.093)	0.547 (0.039)	**0.917 (0.052)**
	GBDT	0.844 (0.040)	0.618 (0.073)	0.616 (0.050)	0.843 (0.034)	0.591 (0.075)	0.658 (0.040)	0.886 (0.041)
	NetLapRLS(L1000)[1]	0.784 (0.035)	0.533 (0.071)	0.529 (0.052)	0.782 (0.027)	0.471 (0.059)	0.638 (0.056)	0.816 (0.028)
	NetLapRLS(PPI)[2]	0.730 (0.042)	0.418 (0.072)	0.472 (0.053)	0.733 (0.039)	0.387 (0.079)	0.626 (0.032)	0.757 (0.047)
	NetLapRLS(combined)[3]	0.796 (0.043)	0.552 (0.074)	0.550 (0.054)	0.797 (0.039)	0.493 (0.087)	0.646 (0.038)	0.833 (0.049)
	EXP2SL(no BPR loss)[4]	0.853 (0.039)	**0.652 (0.070)**	**0.629 (0.054)**	**0.856 (0.056)**	**0.631 (0.095)**	0.639 (0.052)	0.908 (0.077)
	EXP2SL(PPI)[5]	**0.855 (0.040)**	0.650 (0.071)	0.626 (0.047)	0.848 (0.039)	0.602 (0.075)	**0.666 (0.041)**	0.891 (0.050)
	EXP2SL	0.853 (0.035)	0.646 (0.067)	0.625 (0.046)	0.851 (0.040)	0.613 (0.088)	0.650 (0.040)	0.898 (0.055)
A375	LR	0.999 (0.007)	0.999 (0.011)	0.993 (0.015)	0.994 (0.014)	0.990 (0.028)	0.997 (0.003)	0.990 (0.027)
	RF	0.997 (0.007)	0.998 (0.008)	0.992 (0.015)	0.991 (0.015)	0.991 (0.028)	0.994 (0.009)	0.987 (0.028)
	SVM	0.973 (0.010)	0.965 (0.015)	0.950 (0.019)	0.953 (0.018)	0.933 (0.025)	0.977 (0.009)	0.933 (0.024)
	GBDT	0.998 (0.003)	0.999 (0.002)	0.990 (0.008)	0.991 (0.008)	0.983 (0.012)	0.999 (0.005)	0.982 (0.010)
	NetLapRLS(L1000)[1]	0.994 (0.006)	0.996 (0.005)	0.986 (0.013)	0.987 (0.013)	0.987 (0.022)	0.988 (0.007)	0.989 (0.026)
	NetLapRLS(PPI)[2]	0.993 (0.017)	0.995 (0.027)	0.978 (0.023)	0.979 (0.021)	0.973 (0.029)	0.986 (0.032)	0.971 (0.024)
	NetLapRLS(combined)[3]	0.994 (0.007)	0.996 (0.007)	0.980 (0.021)	0.980 (0.017)	0.981 (0.025)	0.983 (0.016)	0.980 (0.021)
	EXP2SL(no BPR loss)[4]	1.000 (0.008)	1.000 (0.010)	1.000 (0.015)	1.000 (0.014)	1.000 (0.021)	1.000 (0.007)	1.000 (0.020)
	EXP2SL(PPI)[5]	1.000 (0.010)	1.000 (0.010)	**1.000 (0.016)**	**1.000 (0.014)**	**1.000 (0.016)**	1.000 (0.021)	**1.000 (0.025)**
	EXP2SL	**1.000 (0.009)**	**1.000 (0.008)**	0.999 (0.014)	0.999 (0.015)	0.998 (0.020)	**1.000 (0.007)**	0.997 (0.022)
HT29	LR	0.958 (0.021)	0.911 (0.037)	**0.876 (0.020)**	**0.945 (0.010)**	**0.917 (0.045)**	0.852 (0.038)	**0.970 (0.015)**
	RF	0.949 (0.018)	0.891 (0.034)	0.846 (0.033)	0.931 (0.016)	0.872 (0.063)	0.849 (0.048)	0.956 (0.023)
	SVM	0.928 (0.019)	0.838 (0.024)	0.821 (0.022)	0.920 (0.009)	0.837 (0.027)	0.836 (0.045)	0.943 (0.009)
	GBDT	0.943 (0.010)	0.884 (0.029)	0.841 (0.028)	0.931 (0.011)	0.904 (0.037)	0.809 (0.041)	0.964 (0.014)
	NetLapRLS(L1000)[1]	0.929 (0.020)	0.857 (0.047)	0.828 (0.050)	0.922 (0.025)	0.858 (0.058)	0.823 (0.037)	0.949 (0.028)
	NetLapRLS(PPI)[2]	0.909 (0.018)	0.830 (0.033)	0.803 (0.033)	0.906 (0.015)	0.824 (0.051)	0.811 (0.037)	0.928 (0.019)
	NetLapRLS(combined)[3]	0.920 (0.016)	0.847 (0.033)	0.822 (0.029)	0.917 (0.016)	0.858 (0.056)	0.816 (0.033)	0.942 (0.019)
	EXP2SL(no BPR loss)[4]	**0.960 (0.020)**	0.913 (0.025)	0.872 (0.013)	0.940 (0.005)	0.891 (0.031)	**0.875 (0.038)**	0.954 (0.013)
	EXP2SL(PPI)[5]	0.959 (0.022)	0.916 (0.039)	0.870 (0.029)	0.942 (0.017)	0.908 (0.072)	0.854 (0.031)	0.965 (0.030)
	EXP2SL	0.960 (0.021)	**0.917 (0.046)**	0.871 (0.038)	0.943 (0.021)	0.912 (0.048)	0.851 (0.038)	0.969 (0.028)

[1] The NetLapRLS method using only the L1000 similarities
[2] The NetLapRLS method using only the PPI similarities
[3] The NetLapRLS method using the combination of L1000 and PPI similarities
[4] The EXP2SL model without the BPR loss
[5] The EXP2SL model with additional PPI information incorporated by a graph convolution module

Table S2. Performance evaluation in three different cell lines under the "split gene" setting, with labels selected by threshold 10%. The mean and standard deviation (in brackets) of each metrics over 10 repeats are shown. The best results for each cell line and each metric are marked in bold.

Dataset	Model name	AUC	AUPR	F1	Accuracy	Precision	Sensitivity	Specificity
A549	LR	0.682 (0.033)	0.423 (0.042)	0.440 (0.029)	0.684 (0.039)	0.401 (0.046)	**0.593 (0.042)**	0.689 (0.060)
	RF	0.721 (0.028)	0.456 (0.039)	0.456 (0.031)	0.735 (0.059)	0.425 (0.051)	0.557 (0.057)	0.765 (0.082)
	SVM	0.701 (0.034)	0.431 (0.044)	0.449 (0.027)	0.732 (0.038)	0.433 (0.045)	0.558 (0.055)	0.762 (0.060)
	GBDT	0.702 (0.032)	0.428 (0.039)	0.439 (0.024)	0.716 (0.044)	0.385 (0.043)	0.560 (0.055)	0.741 (0.065)
	NetLapRLS(L1000)[1]	0.664 (0.030)	0.407 (0.037)	0.438 (0.023)	0.741 (0.053)	0.407 (0.044)	0.507 (0.049)	0.790 (0.076)
	NetLapRLS(PPI)[2]	0.636 (0.033)	0.335 (0.044)	0.386 (0.027)	0.636 (0.031)	0.306 (0.059)	0.581 (0.033)	0.636 (0.044)
	NetLapRLS(combined)[3]	0.668 (0.031)	0.417 (0.042)	0.440 (0.029)	0.751 (0.051)	0.424 (0.053)	0.490 (0.053)	**0.805 (0.076)**
	EXP2SL(no BPR loss)[4]	0.692 (0.035)	0.445 (0.047)	0.450 (0.031)	0.729 (0.032)	0.425 (0.037)	0.545 (0.053)	0.764 (0.051)
	EXP2SL(PPI)[5]	**0.737 (0.030)**	0.484 (0.041)	0.483 (0.028)	0.758 (0.040)	**0.449 (0.052)**	0.568 (0.037)	0.793 (0.061)
	EXP2SL	0.736 (0.033)	**0.486 (0.041)**	**0.485 (0.024)**	**0.762 (0.045)**	0.447 (0.052)	0.565 (0.045)	0.801 (0.068)
A375	LR	0.923 (0.030)	0.871 (0.048)	0.880 (0.029)	0.900 (0.028)	0.848 (0.035)	0.951 (0.028)	0.864 (0.028)
	RF	0.925 (0.020)	0.874 (0.034)	0.882 (0.012)	0.905 (0.018)	0.864 (0.029)	0.940 (0.011)	0.877 (0.022)
	SVM	0.890 (0.044)	0.849 (0.062)	0.859 (0.033)	0.870 (0.034)	0.831 (0.045)	0.935 (0.027)	0.809 (0.050)
	GBDT	0.911 (0.059)	0.868 (0.028)	0.892 (0.032)	0.902 (0.055)	0.868 (0.038)	**0.960 (0.031)**	0.862 (0.077)
	NetLapRLS(L1000)[1]	0.895 (0.033)	0.830 (0.050)	0.859 (0.028)	0.883 (0.029)	0.851 (0.054)	0.898 (0.033)	0.901 (0.060)
	NetLapRLS(PPI)[2]	0.901 (0.046)	0.847 (0.047)	0.866 (0.028)	0.888 (0.050)	0.869 (0.064)	0.895 (0.039)	0.911 (0.144)
	NetLapRLS(combined)[3]	0.898 (0.029)	0.836 (0.047)	0.862 (0.038)	0.885 (0.036)	0.854 (0.044)	0.900 (0.045)	0.904 (0.026)
	EXP2SL(no BPR loss)[4]	0.929 (0.044)	0.892 (0.067)	0.901 (0.047)	0.920 (0.043)	0.887 (0.047)	0.949 (0.030)	0.896 (0.030)
	EXP2SL(PPI)[5]	**0.961 (0.029)**	**0.925 (0.037)**	**0.917 (0.030)**	**0.944 (0.031)**	**0.911 (0.029)**	0.942 (0.036)	**0.935 (0.035)**
	EXP2SL	0.952 (0.033)	0.905 (0.049)	0.900 (0.029)	0.931 (0.031)	0.880 (0.047)	0.947 (0.031)	0.918 (0.049)
HT29	LR	0.796 (0.035)	0.566 (0.059)	0.635 (0.066)	0.798 (0.031)	0.597 (0.067)	0.761 (0.085)	0.794 (0.041)
	RF	0.842 (0.026)	0.592 (0.038)	0.659 (0.037)	0.827 (0.028)	0.611 (0.036)	0.768 (0.052)	0.833 (0.035)
	SVM	0.815 (0.044)	0.549 (0.042)	0.639 (0.045)	0.791 (0.032)	0.552 (0.063)	**0.851 (0.063)**	0.760 (0.045)
	GBDT	0.791 (0.029)	0.537 (0.039)	0.584 (0.039)	0.763 (0.039)	0.534 (0.067)	0.738 (0.035)	0.757 (0.052)
	NetLapRLS(L1000)[1]	0.798 (0.039)	0.592 (0.071)	0.645 (0.040)	0.821 (0.054)	0.609 (0.084)	0.736 (0.057)	**0.843 (0.103)**
	NetLapRLS(PPI)[2]	0.756 (0.035)	0.522 (0.042)	0.586 (0.027)	0.778 (0.031)	0.554 (0.026)	0.708 (0.075)	0.790 (0.056)
	NetLapRLS(combined)[3]	0.791 (0.032)	0.585 (0.046)	0.626 (0.019)	0.809 (0.023)	0.603 (0.035)	0.719 (0.051)	0.830 (0.039)
	EXP2SL(no BPR loss)[4]	0.816 (0.031)	0.600 (0.066)	0.672 (0.044)	0.824 (0.039)	**0.646 (0.068)**	0.770 (0.084)	0.828 (0.059)
	EXP2SL(PPI)[5]	**0.852 (0.033)**	0.642 (0.042)	**0.680 (0.039)**	**0.836 (0.041)**	0.630 (0.047)	0.796 (0.034)	0.838 (0.065)
	EXP2SL	0.851 (0.037)	**0.643 (0.045)**	0.677 (0.034)	0.836 (0.027)	0.624 (0.060)	0.797 (0.080)	0.834 (0.049)

[1] The NetLapRLS method using only the L1000 similarities
[2] The NetLapRLS method using only the PPI similarities
[3] The NetLapRLS method using the combination of L1000 and PPI similarities
[4] The EXP2SL model without the BPR loss
[5] The EXP2SL model with additional PPI information incorporated by a graph convolution module

Table S3. Top 10 over-repensented GO biological process annotations among the top 50 important genes selected for cell line HT29, calculated using the WebGestalt server (Liao et al., 2019).

Gene Set	Description	Size	Expect	Ratio	P Value	FDR
GO:0048661	positive regulation of smooth muscle cell proliferation	82	0.21157	28.360	6.2110e-8	0.00020357
GO:0000302	response to reactive oxygen species	215	0.55472	14.422	6.6248e-8	0.00020357
GO:0042981	regulation of apoptotic process	1517	3.9140	4.3434	7.8291e-8	0.00020357
GO:0043067	regulation of programmed cell death	1531	3.9501	4.3036	8.9571e-8	0.00020357
GO:1901701	cellular response to oxygen-containing compound	1074	2.7710	5.0523	2.6263e-7	0.00032465
GO:0010941	regulation of cell death	1649	4.2546	3.9957	2.6354e-7	0.00032465
GO:0043065	positive regulation of apoptotic process	606	1.5635	7.0353	2.6751e-7	0.00032465
GO:0043068	positive regulation of programmed cell death	610	1.5739	6.9892	2.8569e-7	0.00032465
GO:0006915	apoptotic process	1912	4.9332	3.6488	3.9245e-7	0.00039642
GO:0034599	cellular response to oxidative stress	278	0.71727	11.153	4.7381e-7	0.00043074

Table S4. Top 10 over-repensented GO biological process annotations among the top 50 important genes selected for cell line A375, calculated using the WebGestalt server (Liao et al., 2019).

Gene Set	Description	Size	Expect	Ratio	P Value	FDR
GO:0071260	cellular response to mechanical stimulus	78	0.22933	21.803	0.0000032011	0.019405
GO:0031400	negative regulation of protein modification process	560	1.6465	6.0736	0.0000042691	0.019405
GO:0051254	positive regulation of RNA metabolic process	1668	4.9041	3.2626	0.000012751	0.028595
GO:0001933	negative regulation of protein phosphorylation	385	1.1319	7.0675	0.000014854	0.028595
GO:0009057	macromolecule catabolic process	1338	3.9339	3.5588	0.000020042	0.028595
GO:0032269	negative regulation of cellular protein metabolic process	983	2.8901	4.1521	0.000020061	0.028595
GO:0007568	aging	303	0.89086	7.8576	0.000027348	0.028595
GO:0042326	negative regulation of phosphorylation	423	1.2437	6.4326	0.000029192	0.028595
GO:0009612	response to mechanical stimulus	207	0.60860	9.8586	0.000030597	0.028595
GO:0051248	negative regulation of protein metabolic process	1044	3.0695	3.9095	0.000036402	0.028595

Table S5. Top 10 over-repensented GO biological process annotations among the top 50 important genes selected for cell line A549, calculated using the WebGestalt server (Liao et al., 2019).

Gene Set	Description	Size	Expect	Ratio	P Value	FDR
GO:0043535	regulation of blood vessel endothelial cell migration	88	0.26401	18.939	0.0000064300	0.058455
GO:0043534	blood vessel endothelial cell migration	112	0.33601	14.880	0.000020861	0.094825
GO:0071216	cellular response to biotic stimulus	222	0.66603	9.0086	0.000050863	0.10038
GO:0033673	negative regulation of kinase activity	237	0.71103	8.4385	0.000073096	0.10038
GO:0009607	response to biotic stimulus	926	2.7781	3.9595	0.000074342	0.10038
GO:0010594	regulation of endothelial cell migration	151	0.45302	11.037	0.000087157	0.10038
GO:0010243	response to organonitrogen compound	945	2.8351	3.8799	0.000089213	0.10038
GO:0030336	negative regulation of cell migration	252	0.75603	7.9362	0.00010249	0.10038
GO:1901700	response to oxygen-containing compound	1556	4.6682	2.9990	0.00013465	0.10038
GO:0051348	negative regulation of transferase activity	266	0.79803	7.5185	0.00013778	0.10038

Table S6. Top 10 over-repensented KEGG pathways among the top 50 important genes selected for cell line HT29, calculated using the WebGestalt server (Liao et al., 2019).

Gene Set	Description	Size	Expect	Ratio	P Value	FDR
hsa05212	Pancreatic cancer	75	0.30125	16.598	0.000010453	0.0024818
hsa04012	ErbB signaling pathway	85	0.34141	14.645	0.000019317	0.0024818
hsa05200	Pathways in cancer	526	2.1127	4.7332	0.000022839	0.0024818
hsa04510	Focal adhesion	199	0.79930	7.5065	0.00011544	0.0073771
hsa05223	Non-small cell lung cancer	66	0.26510	15.089	0.00012823	0.0073771
hsa05214	Glioma	71	0.28518	14.026	0.00017048	0.0073771
hsa05218	Melanoma	72	0.28920	13.831	0.00018001	0.0073771
hsa04915	Estrogen signaling pathway	137	0.55027	9.0864	0.00019014	0.0073771
hsa05220	Chronic myeloid leukemia	76	0.30526	13.104	0.00022199	0.0073771
hsa05163	Human cytomegalovirus infection	225	0.90374	6.6391	0.00022629	0.0073771

Table S7. Top 10 over-repensented KEGG pathways among the top 50 important genes selected for cell line A375, calculated using the WebGestalt server (Liao et al., 2019)

Gene Set	Description	Size	Expect	Ratio	P Value	FDR
hsa04218	Cellular senescence	160	0.53555	13.071	6.3096e-7	0.00020569
hsa05170	Human immunodeficiency virus 1 infection	212	0.70960	8.4555	0.000054914	0.0089510
hsa04620	Toll-like receptor signaling pathway	104	0.34811	11.491	0.00035831	0.038936
hsa05145	Toxoplasmosis	113	0.37823	10.576	0.00049166	0.040070
hsa04110	Cell cycle	124	0.41505	9.6374	0.00069878	0.045353
hsa04926	Relaxin signaling pathway	130	0.43513	9.1926	0.00083471	0.045353
hsa05166	Human T-cell leukemia virus 1 infection	255	0.85353	5.8580	0.0013522	0.062974
hsa04115	p53 signaling pathway	72	0.24100	12.448	0.0016967	0.069124
hsa05212	Pancreatic cancer	75	0.25104	11.950	0.0019083	0.069124
hsa00900	Terpenoid backbone biosynthesis	22	0.073638	27.16	0.0023849	0.077747

Table S8. Top 10 over-repensented KEGG pathways among the top 50 important genes selected for cell line A549, calculated using the WebGestalt server (Liao et al., 2019)

Gene Set	Description	Size	Expect	Ratio	P Value	FDR
hsa04919	Thyroid hormone signaling pathway	116	0.52805	7.5751	0.0017879	0.18261
hsa04110	Cell cycle	124	0.56447	7.0863	0.0022831	0.18261
hsa05160	Hepatitis C	131	0.59633	6.7077	0.0027882	0.18261
hsa05162	Measles	132	0.60088	6.6569	0.0028661	0.18261
hsa05211	Renal cell carcinoma	69	0.31410	9.5512	0.0036789	0.18261
hsa05165	Human papillomavirus infection	339	1.5432	3.8881	0.0038256	0.18261
hsa05161	Hepatitis B	144	0.65551	6.1021	0.0039211	0.18261
hsa05220	Chronic myeloid leukemia	76	0.34596	8.6714	0.0048304	0.19684
hsa04218	Cellular senescence	160	0.72834	5.4919	0.0057019	0.20653
hsa04012	ErbB signaling pathway	85	0.38693	7.7533	0.0066003	0.21517

REFERENCES

Lei, T., Zhang, Y., Wang, S. I., Dai, H., and Artzi, Y. (2018). Simple recurrent units for highly parallelizable recurrence. In *Proceedings of the 2018 Conference on Empirical Methods in Natural Language Processing*. 4470–4481

Liao, Y., Wang, J., Jaehnig, E. J., Shi, Z., and Zhang, B. (2019). Webgestalt 2019: gene set analysis toolkit with revamped UIs and APIs. *Nucleic acids research*

Subramanian, A., Narayan, R., Corsello, S. M., Peck, D. D., Natoli, T. E., Lu, X., et al. (2017). A next generation connectivity map: L1000 platform and the first 1,000,000 profiles. *Cell* 171, 1437–1452

Szklarczyk, D., Franceschini, A., Wyder, S., Forslund, K., Heller, D., Huerta-Cepas, J., et al. (2014). STRING v10: protein–protein interaction networks, integrated over the tree of life. *Nucleic acids research* 43, D447–D452
